# Legalization dilemma and solution of China's professional sports league governance

**DOI:** 10.3389/fspor.2026.1748804

**Published:** 2026-02-02

**Authors:** Quansheng Wang, Ziqing Zhang, Guoqing Han

**Affiliations:** Discipline Inspection and Supervision School, Shandong University, Weihai, China

**Keywords:** professional sports league, rule of law, separation of administration andmanagement, sports governance, sports law

## Abstract

This paper takes the frequent dissolution of clubs and wage arrears of players in China's professional sports leagues in recent years as a starting point, revealing the deep-seated governance crisis under its prosperous appearance. By combining the progress of Chinese professional sports in terms of policy-driven and legal evolution, the article focuses on analyzing its legalization dilemma in the four core areas of governance structure, property rights system, regulatory system, and player mobility. On this basis, drawing on mature European and American experience and combining with China's national conditions, it puts forward a systematic legal path with “charterization” governance as the core, clarifying the status of legal persons, strengthening the application of law, and constructing a special litigation mechanism to provide theoretical reference for promoting the high-quality development of China's professional sports leagues.

## 1 Introduction

Since 2023, a number of events that have caused widespread social concern have erupted in the field of Chinese professional sports leagues (1). In the Chinese Super League, the former champion team Guangzhou Evergrande has fallen into a crisis of dissolution due to the break of the capital chain, and the players' salaries have not been paid on time for as long as half a year. Some players have been forced to publicly demand wages through social media; a CBA league club was exposed to the problem of “yin and yang contracts”, and the actual income of players was very different from the amount of the filed contract, which not only violated the principle of fair competition in the league, but also triggered public doubts about the integrity system of professional sports (2). These events are not isolated but a concentrated outbreak of governance contradictions in the rapid development of China's professional sports leagues, reflecting the deep crisis under the prosperous appearance of the league (3).

After decades of development, China's professional sports leagues have gradually moved from the initial amateur events to marketization and specialization (4). The attention and commercial value of the Chinese Super League, CBA, and other leagues have been continuously improved, becoming an important part of the sports industry (5). However, with the expansion of the league scale and the diversification of interest groups, problems such as the imperfect governance system and the lack of legal protection have become increasingly prominent. Problems such as the cross-cutting of powers and responsibilities between industry associations and league companies, the ambiguity of property rights, the absence of regulatory mechanisms, and the inadequate protection of players' rights not only restrict the high-quality development of the league but also affect the social credibility of professional sports (6). In the background of building a sports power, the professional sports league, as an important carrier for the development of sports, its governance level is directly related to the upgrading of the sports industry and the dissemination of sports culture. Therefore, it is of great theoretical value and practical significance to deeply analyze the legalization dilemma faced by China's professional sports leagues and explore a governance path that conforms to China's national conditions.

## 2 Theoretical analytical framework

To systematically dissect the deep-seated contradictions in the governance of China's professional sports leagues, this paper draws upon the classic theoretical framework for sports governance research proposed by Henry and Lee (7). This framework divides sports governance practices into three interrelated core domains: organizational governance, systemic governance, and political governance. This theory provides a structured analytical perspective for understanding the legalization dilemmas faced by China's professional sports leagues, helping to reveal the inherent logic and multidimensional characteristics of these challenges.

### 2.1 Organizational governance

Organizational governance focuses on the internal structure, decision-making processes, and accountability mechanisms within sports organizations (8). It examines how entities such as industry associations, league companies, and clubs design their governance frameworks, define authority boundaries, and ensure transparent decision-making and accountability (9). In the Chinese context, the challenges of organizational governance are epitomized by the incomplete implementation of the “separation of management and operations” reform (e.g., excessive control by industry associations over league companies), imperfect corporate governance structures within league companies (e.g., lack of independent decision-making authority), weak influence of clubs within governance structures, and unfair revenue distribution coupled with short-term investment tendencies stemming from ambiguous property rights (10, 11). These are precisely the governance structure contradictions and issues of unclear property rights systems that will be analyzed in detail later in this paper.

### 2.2 Systemic governance

Systemic governance focuses on the interactive relationships among the constituent elements of the entire sports system (organizations, rules, participants, resources), the coordination of regulatory frameworks, and the effectiveness of external oversight (12). It emphasizes the soundness of rules (such as league constitutions, transfer regulations, and financial fair play legislation), consistent enforcement, and the synergistic operation of internal and external oversight mechanisms (such as disciplinary committees, arbitration, and judicial intervention) (13). The systemic governance challenges in China's professional sports leagues are particularly evident in: inadequate youth development compensation and player mobility rules that conflict with international standards; dual regulatory failures within and outside the industry (insufficient independence of internal bodies and limited external judicial recourse); and difficulties in applying universal laws like the Anti-Monopoly Law and Labor Contract Law to the sports sector (14). This corresponds to the issues of regulatory gaps and insufficient player rights protection that this paper will explore in depth.

### 2.3 Political governance

Political governance involves the interaction between sport and the broader political, legal, and social environment, particularly concerning policy formulation, legal frameworks, and the role and influence of government/public authorities in sports governance (15). It examines how national policies guide sports development, how legal systems provide safeguards for sport, and the balance between administrative power and market autonomy (16). The development of China's professional sports leagues has been distinctly policy-driven (e.g., a series of State Council and General Administration of Sport documents promoting market-oriented reforms), and the 2022 revision of the Sports Law marked a breakthrough in professional sports legislation. However, challenges at the political governance level include: excessive administrative intervention in policy implementation coupled with a lack of market coordination mechanisms; legal provisions (such as the professional sports chapter of the Sports Law) being highly principled while supporting regulations lag; and the boundaries of authority and relationships of interest between the state, associations, leagues, and clubs not yet being fully clarified through law and institutions (17, 18). This constitutes the key background for understanding China's professional sports development model and the obstacles to its legalization.

Employing Henry and Lee's three-dimensional governance framework, this paper clearly attributes the complex legalization challenges facing China's professional sports leagues to three key factors: organizational-level imbalances in the structure of authority and responsibility, systemic-level failures in rules and regulation, and political-level tensions between policy/legal frameworks and market practices. This theoretical perspective not only provides a rigorous logical starting point for subsequent analysis but also lays the theoretical foundation for exploring systemic solutions.

## 3 Development of China's professional sports league

The birth and development of China's professional sports league is not the result of pure market spontaneity, but the product of a strong national policy, driven by and gradual improvement of the legal system.

### 3.1 Policy-driven: promoting marketization and specialization reform

In recent years, the state has issued a series of policy documents to provide policy support for the development of professional sports leagues and promote the transformation of leagues to marketization and specialization (see Table 1).

**Table 1:** Documents of support for policies related to the development of professional sports leagues issued by China.

No	File name	Formulating entity	Effective date	Main content
1	Several Opinions on Accelerating the Development of the Sports Industry and Promoting Sports Consumption	State Council	October 20, 2014	For the first time at the national level, it proposed “Promoting the reform of professional sports and encouraging the establishment of professional leagues; improving the grading system of professional leagues”.
2	Overall Plan for the Reform and Development of Chinese Football	General Office of the State Council	March 8, 2015	Clearly stated “Adjust and establish the Professional League Council (Chinese Super League Company) to separate management from operation; establish a market-oriented operation mechanism for copyright and broadcast rights of the league”.
3	Sports Development “13th Five-Year Plan”	General Administration of Sport of China	May 5, 2016	Proposed “Improve the football competition system and professional league system, improve the corporate governance structure of professional clubs, and accelerate the construction of a modern enterprise system”.
4	Guiding Opinions on Accelerating the Development of the Sports Competition Performance Industry	General Office of the State Council	December 21, 2018	Quantified goals: By 2025, the total scale of the sports competition performance industry will reach 2 trillion yuan; create 100 high-quality events with greater popularity; support professional alliances to grow stronger.
5	Notice on Issuing the Outline for Building a Leading Sports Nation	General Office of the State Council	August 10, 2019	Promote the innovative development of professional sports, expand the social influence of professional sports, and improve the maturity and standardization of professional sports.
6	Opinions on Promoting National Fitness and Sports Consumption to Promote High-Quality Development of the Sports Industry	General Office of the State Council	September 4, 2019	Article 22 Special Funds: “Focus on developing existing professional leagues, encourage sports programs with the conditions to hold professional events, and rationally construct a grading system for professional leagues; support the establishment of various professional alliances”.
7	“14th Five-Year Plan” for Sports Development	General Administration of Sport of China	October 8, 2021	Special chapter deployment “Professional Sports Development Project”: Promote modernization of the professional league governance system and improve professional club access, youth training, financial fairness, player mobility, and other systems; create 3 to 5 world-class professional league brands.
8	Implementation Opinions on the Reform and Development of Chinese Youth Football (Sports Development	General Administration of Sport of China and 12 other ministries and commissions	2024 Year 2 Month 23 Day	Clearly, “Improve the youth league system: campus leagues, social youth training leagues and professional club elite leagues are interconnected; By 2030, a pyramid of national youth football leagues will be built with vertical and horizontal connections”.

Under the guidance of policies, the marketization of China's professional sports leagues has gradually increased. On the one hand, breakthroughs have been made in the commercial development of the leagues. The Chinese Super League (CSL) has reached high-value sponsorship agreements with many companies, and the CBA League has achieved revenue growth through copyright sales, advertising, and other methods. The enthusiasm of market players to participate in league operations has continued to increase (19). However, professionalization of leagues has been accelerated by introducing foreign coaches, players, and professional management talents, optimizing event organization processes, and improving event quality and viewing experience (20). For example, the CSL has gradually improved the player transfer system and the salary cap system, and the CBA League has established a draft system and a player development league (21). These measures have, to some extent, promoted the leagues towards standardization. However, it should also be noted that reforms driven by policies still have the problem of “excessive administrative intervention”. Some policies, in the process of implementation, lack supporting measures and market coordination mechanisms, failing to fully leverage the decisive role of the market in resource allocation, which affects the in-depth promotion of the market-oriented reform of the leagues (22).

### 3.2 Legal evolution: revision of the sports Law and breakthroughs in professional sports legislation

The revised “Sports Law of the People's Republic of China” in 2022 added a chapter on “Professional Sports,” which is the first time in China that the basic sports law at the national level has specifically stipulated professional sports, filling the gap in professional sports legislation. The chapter clarifies that “the state promotes the development of professional sports, encourages the establishment of professional sports leagues, improves the corporate governance structure of professional sports clubs, and regulates the operation of professional sports clubs”. At the same time, it makes principled provisions on issues such as registration, transfer, salary, and rights protection of professional athletes, providing a legal basis for the governance of professional sports leagues (20).

However, the provisions on professional sports in the “Sports Law” still have limitations. From the perspective of content, the relevant clauses are mostly principled provisions and lack specific implementation rules. For example, the establishment conditions and operating mechanisms of the “professional sports league” and the specific requirements of the “corporate governance structure of clubs” have not yet been clarified, making it difficult to directly apply the legal clauses in practice. From the perspective of the scope of adjustment, the “Sports Law” does not stipulate key issues such as the ownership of league copyrights, the distribution of commercial development revenue, and the connection between sports arbitration and judicial remedies, and cannot fully cover the complex contradictions in league governance. From the perspective of the legal level, the “Sports Law,” as a basic law, is difficult to regulate the specific issues of professional sports leagues in detail and requires supporting administrative regulations, departmental rules, and industry rules to supplement it, but the relevant supporting legislation is still relatively lagging, which restricts the guarantee role of the law in league governance (2, 23).

## 4 The legal challenges faced by China’s professional sports leagues

Despite significant progress in policies and laws, league governance still faces multiple legal challenges in practice.

### 4.1 Contradictions in the governance structure of professional sports leagues

“Separation of management and operation” is the core issue of China's professional sports reform, which aims to separate the administrative management functions of industry associations from the commercial operation functions of leagues (24). However, years of reform practice have shown that this separation is not thorough. On the one hand, industry associations have nominally withdrawn from the direct operation of the leagues and established independent league companies, but the associations often maintain absolute control over the league companies through methods such as “veto power” and personnel appointment and removal rights (25). On the other hand, league companies, as nominal market entities, still need to obey the associations for their major decisions and lack true independent operating rights. This “separation in form but not in substance” governance structure has led to blurred boundaries of rights, responsibilities, and interests among industry associations, league companies, and clubs. The association is both a “referee” and an “athlete,” both formulating rules and participating in the distribution of benefits, making it difficult to ensure the neutrality and fairness of decisions. Clubs, as true participants and contributors to the league, have weak voices in the governance structure, and their legitimate rights and interests are often violated, triggering continuous contradictions and conflicts (26).

### 4.2 Vague property rights system of professional sports leagues

Clear property rights are the cornerstone of a market economy. In China's professional sports leagues, the ambiguity of the property rights system is a fatal weakness. Who owns the core assets of the league, such as event copyrights, commercial development rights, naming rights, and derivative development rights? Is it the league company representing all clubs, the association as the industry manager, or the state that owns the intangible assets? This fundamental issue has never been clearly defined (27). Unclear property rights directly lead to an unfair and chaotic distribution of income. For example, how to distribute the sky-high broadcasting revenue is often decided by the association, and clubs lack bargaining power. This uncertainty also seriously affects investors' confidence because the value of club equity largely depends on its share of rights and interests in the league's core assets. Without stable property rights expectations, it is difficult for investors to make long-term plans, and the equity structure of clubs is therefore turbulent, with “short-sighted operation” and “speculative behavior” being common (28).

### 4.3 Absence of a regulatory system for professional sports leagues

An effective regulatory system should be an organic combination of internal self-discipline and external other-discipline. At present, there is a clear absence of a regulatory system for professional sports in China (29). Internally, the disciplinary committee and arbitration committee under the industry association are the main institutions for handling disputes, but their independence and impartiality are highly questioned because they are essentially still “internal departments” of the association. Externally, the channels for judicial relief are not smooth. For a long time, the court system has generally taken a cautious approach to internal disputes in the sports industry, often refusing to accept or dismissing lawsuits because they “belong to the scope of industry autonomy,” which has led to the so-called “judicial relief vacuum”. In addition, the application of the Anti-Monopoly Law in the sports field also faces difficulties (30). Whether certain behaviors of industry associations, such as restricting player transfers and unifying sponsor pricing, constitute monopoly behaviors lacks a clear judicial definition and effective regulation. This dual failure of internal and external supervision makes it difficult to punish league violations in a timely and fair manner, which seriously weakens the authority of the law (22).

### 4.4 Insufficient protection of youth training compensation and player mobility rights

Youth training is the foundation of professional sports, and player mobility is the embodiment of market vitality. In these two links, there are significant problems in China's rule system. The first is the imperfect youth training compensation mechanism. Compared with FIFA's mature joint mechanism compensation and training compensation regulations, the relevant domestic compensation standards are low and difficult to implement, leading to frequent “poaching” phenomena, which seriously dampen the enthusiasm of clubs to invest in youth training (31). Secondly, domestic transfer rules often conflict with international rules, especially in terms of player identity recognition and contract dispute resolution, lacking a clear and unified coordination mechanism (32). Finally, players, as the core factors of production in the league, have insufficient protection of their rights and interests. Wage arrears are repeatedly prohibited, contract disputes occur frequently, and players are often in a weak position in the face of powerful clubs and associations, lacking effective channels for safeguarding their rights (33). The labor supervision department has insufficient understanding of the particularities of the sports field, and internal arbitration in the industry is difficult to be completely fair, resulting in the basic rights granted to players by the Labor Contract Law often being hollowed out in the field of professional sports (23).

## 5 Legal thinking on the governance of China's professional sports leagues

### 5.1 Building a “chartered” governance model

Mature European and American professional sports leagues provide us with valuable experience. Its core characteristic is “chartered governance”. The league is an entity jointly owned and governed by the clubs. The league charter is like a “constitution”, stating the purpose, organizational structure, decision-making mechanism, revenue distribution method, and the rights and obligations of all members. In this model, the league implements professional operations under the leadership of the board of directors. The league president is responsible to the board of directors. The industry association mainly plays the role of macro guidance, national team construction, and industry supervision, and does not directly interfere in the league's commercial operations (25).

Drawing on European and American experience and combining it with China's national conditions, China's professional sports leagues should build a “chartered” governance model, with the league charter as the core, clarifying the rights, responsibilities, and interests of all parties, such as industry associations, league companies, and clubs (34). First, formulate a unified league charter and use the charter as the “fundamental law” of league governance, clarifying the purpose, organizational structure, decision-making mechanism, revenue distribution, dispute resolution, and other core content to ensure that league governance has rules to follow. Second, improve the organizational structure of the league and establish a governance system of “industry association supervision + league company operation + club participation”. The industry association is responsible for macro supervision, policy guidance and industry self-discipline of the league, and shall not directly interfere with league daily operations; the league company, as an independent legal entity, is responsible for the commercial development, event organization, brand building, and other operational work of the league, and has independent decision-making power and management rights; the clubs, as the main participants of the league, participate in league decision making through the league's board of directors, shareholders' meetings, and other forms, and enjoy equal say in revenue distribution, rule making, and other aspects. Finally, establish and improve the league's decision-making mechanism, implement “democratic centralism” decision-making, and major matters involving the development of the league must be voted on by the league's board of directors or the club representatives' meeting to ensure scientific and fair decision-making (35).

### 5.2 Clarify the legal entity status of professional sports leagues

Clarifying the legal entity status of the league and improving corporatized operation are key to realizing the market-oriented and professional governance of professional sports leagues (33). At present, Chinese professional sports league companies have problems such as an imperfect corporate governance structure and insufficient independence, which need to be improved in the following aspects:

First, clarify the legal entity status of the league company and achieve complete separation of the league company from the industry association. Through equity reform, reduce the proportion of shares held by the industry association in the league company, introduce social capital and club investment, and make the league company an independent legal entity jointly held by multiple entities. The industry association no longer directly participates in the league company's business decisions, but only performs macromanagement functions by formulating industry rules and implementing supervision to ensure the league company's independent operation (36).

Secondly, improve the corporate governance structure of the league company and establish a standardized modern enterprise system. The league company should establish a shareholders' meeting, board of directors, board of supervisors, and management, clarify the boundaries of power and responsibility of each institution, and form a check-and-balance mechanism of “shareholders' meeting decision-making, board of directors execution, board of supervisors supervision, and management operation”. At the same time, introduce an independent director system, invite experts from the sports industry, legal experts, financial experts, etc., to serve as independent directors, and improve the professionalism and impartiality of the board of directors' decisions (37).

Finally, optimize the league company's operating mechanism and improve commercial development capabilities and service levels. The league company should focus on the development of the event IP, expand commercial fields such as copyright sales, advertising, ticket sales, and derivative development, and increase the commercial value of the league; establish a scientific revenue distribution mechanism, reasonably determine the revenue distribution ratio based on the club's contribution, competition results, youth training investment, and other factors, and protect the legitimate rights and interests of the club (38).

### 5.3 Strengthen the application of law and improve the special sports litigation mechanism

Strengthening the application of laws such as the Anti-Monopoly Law and the Labor Contract Law in the field of professional sports and establishing a special sports litigation mechanism are important ways to solve the lack of league supervision and protect the rights and interests of the parties (39).

In terms of the application of the antimonopoly law, special antimonopoly application rules should be formulated in combination with the particularity of professional sports leagues. First, add a “Special Clause for Professional Sports” to the supporting regulations of the Anti-Monopoly Law to clarify the identification standards and exemption circumstances for monopoly behaviors in professional sports leagues. Second, establish a coordinated supervision mechanism between antimonopoly law enforcement agencies and sports administrative departments, conduct regular antimonopoly reviews of professional sports leagues, and promptly discover and correct monopoly behaviors (40).

In terms of the application of the labor contract law, it is necessary to strengthen the standardization and supervision of players' labor contracts and protect the legitimate rights and interests of players. On the one hand, clarify the necessary clauses of the player's labor contract, require the club to sign a written labor contract with the player, and clarify the salary standard, payment method, contract period, liability for breach of contract, etc., and strictly prohibit the signing of “yin-yang contracts” and “blank contracts”; on the other hand, establish a player labor contract filing system, the league management agency, and the labor administrative department should review the player labor contract for filing, and contracts that violate laws and league rules will not be filed, and the club will be ordered to rectify within a time limit (41).

More importantly, we should draw on international experience and establish an independent and professional “special sports litigation mechanism”. This could be the establishment of a special sports court within the existing court system or the establishment of a sports arbitration court that is truly independent of the industry association and has the effect of final adjudication. The mechanism should be equipped with professionals who understand both law and sports, responsible for handling various sports disputes, realizing the effective connection between industry self-government and judicial relief, and providing all market players with a predictable and fair final remedy channel (42).

## 6 Conclusion

The rule of law in the governance of professional sports leagues is the only way to achieve high-quality development of the sports industry. Only with the guarantee of law, clarifying the relationship of rights, responsibilities, and interests of all parties such as industry associations, league companies, clubs, and players, can the league governance system be straightened out and interest conflicts be resolved; only through legal norms on property rights attribution, market competition, and labor contracts, can the order of fair competition in the league be maintained and the legitimate rights and interests of market players and players be protected; only by establishing and improving the special sports litigation mechanism can the channels for resolving disputes be unblocked, and the efficiency of governance be improved.

In the future, with the improvement of supporting regulations for the “Sports Law,” the construction of a “charter-based” governance model, the promotion of league corporatization, and the establishment of a special sports litigation mechanism, the legal guarantee for China's professional sports leagues will be continuously strengthened, and the level of governance will gradually improve. However, the legal construction of the league is a long-term task that requires the joint efforts of the government, sports administrative departments, league management organizations, clubs, players, and all sectors of society to continuously explore and improve the governance path of professional sports leagues that conforms to China's national conditions in practice, promote the sustainable development of the league, and inject more energy into the construction of a sports powerhouse.
